# Editorial: Omics-based plant traits improvements under destructive stresses

**DOI:** 10.3389/fpls.2024.1356798

**Published:** 2024-01-30

**Authors:** Elsayed Nishawy, Yunpeng Cao, Mohamed Ewas, Mohamed Hamdy Amar, Ibrahim Eid Elesawi

**Affiliations:** ^1^ Laboratory of Genomics and Genome Editing, Department of Genetics, Desert Research Center, Cairo, Egypt; ^2^ CAS Key Laboratory of Plant Germplasm Enhancement and Specialty Agriculture, Wuhan Botanical Garden, Chinese Academy of Sciences (CAS), Wuhan, China; ^3^ Agricultural Biochemistry Department, Faculty of Agriculture, Zagazig University, Zagazig, Egypt

**Keywords:** omics, genomics, functional characterization, plant traits improvements, destructive stresses

## Introduction

The Functional and Applied Plant Genomics section in the journal *Frontiers in Plant Science* chiefly publishes research exploring the mechanism of plant trait improvements under destructive stresses. This Research Topic aims to shed comprehensive light on the mechanism underlying plant responses to stresses at both biological and transcriptional levels. Climate change is one of the most significant threats to current and future global food security, exacerbating destructive stresses and posing severe challenges to agriculture. This includes the reduction and degradation of arable land, loss of plant biodiversity, altesatron of plant traits, and consequent impacts on plant yields. Such consequences have far-reaching effects on global food production and security. In recent times, the rapid development of omics-based technologies, such as transcriptomics and metabolomics, along with functional characterization approaches, has illuminated our understanding of the biological and molecular mechanisms of the plant responses to abiotic stresses such as cold, heat, salinity, and drought. Generating extensive ‘omic data’ is essential to draw a clear vision for plant traits improvement to cope with destructive stresses.

The Research Topic focuses on the understanding of the response mechanisms of plants to environmental stresses through the integration of various ‘omics approaches. The topic was planned to address the mechanisms governing plant responses to these destructive stresses through (I) Releasing novel plant transcriptomes, genomes, proteomes, and metabolomes to identify pathways related to stress tolerance; (II) Functional characterization research on stress-responsive genes; (III) investigating plant diversity, plant breeding using omics approaches and conventional comparative analysis methods of genomics, metabolomics, transcriptomics, and proteomics; (IV) Exploring ‘omics-related traits such as transcriptome-traits, genome-traits, metabolome- traits, and proteome-traits.

Earlier studies reported that transcription factors regulate plant development and stress responses. Li et al. systemically analyzed R2R3-MYB transcription factors in tobacco to understand the role of these genes. The initial examination focused on NtMYB102, revealing that the expression of the NtMYB102 gene is induced in response to salt treatments. Notably, the functional characterization of NtMYB102 demonstrated an augmentation of salt stress tolerance and drought resistance in tobacco.

The plant cell-to-cell signaling network signal is essential in regulating development, growth, as well as tolerance to changes in environment. Plant small secreted peptides (SSPs) serve as vital regulators across a broad spectrum of processes, spanning from germination to flowering and encompassing various developmental stages such as vascular development, meristem maintenance, root growth, pollen tubes growth, casparian strip formation, floral organ abscission, stomatal development, senescence, defense response, and stress acclimation. In a previous study, Tian et al. identified thousands of putative SSPs within the wheat genome. Through a thorough analysis of RNA-seq data, they observed significant up-regulation of hundreds of TaSSP-encoding genes under abiotic stresses. Additionally, the novel TaSSP family CRP8CI and DYY were characterized. Application of exogenous TaCEPID peptide improved the salinity and drought tolerance in wheat plants, demonstrating the potential roles of SSPs in controlling stress responses in wheat.

Globally, Maize (Zea mays L.) is one of the main cereal crops, yet its yield faces reduction worldwide due to the escalating frequency of severe heat events, attributed to the negative impacts of climate change. In a recent study, Chen et al. treated LH150 and PF5411-1 maize inbred lines with alterations in heat tolerance at kernel development stage, and they used transcriptome analysis to explore and discover novel genes and pathways that regulated in response to heat tolerance. Interestingly, the response of LH150 and PF5411-1 to heat pressure resulted in hundreds of up- and down-regulated genes with tenth of putative transcription factors (TFs) under heat stress conditions compared to the control groups. Combined with protein and metabolite data, seven genes involved in several key pathways were highly associated with the heat tolerance of maize kernels. Firstly, a number of small heat shock protein (sHSP) genes were involved in protein processing in endoplasmic reticulum pathway, participating in the process of ER-associated degradation (ERAD). Secondly, flavonoid 3’,5’-hydroxylase (Zm00001d047424) catalyzed the synthesis of myricetin in the myricetin biosynthesis pathway. Thirdly, the raffinose synthase encoding gene (Zm00001d019163) controlled the synthesis of raffinose, and improves the heat resistance of maize kernels. In addition, up-regulated ethylene signaling pathway genes may be associated with heat tolerance of maize kernels. Taken together, these results provide the basis for a better understanding of heat resistance response in maize for further genetics improvements.

*Dendrobium huoshanense* is an herbal plant with an important status in traditional Chinese medicine. The KNOX gene, known for its vital role in responding to different environmental stresses as well as plant growth and development, has been extensively studied in maize and Arabidopsis. However, there is limited research on the identification and expression analysis of KNOX gene family in Dendrobium. Li et al. reported that promoter analysis showed numerous cis-acting elements associated with abiotic stress responses, plant growth, plant development, and hormones. These findings establish the basis for further investigation into the role and mechanisms of the KNOX gene in Dendrobium plants for further plant traits improvements.

## Perspective

In conclusion, this Research Topic lays the foundation for further examination of plant trait regulation under environmental stresses such as salinity, drought, and heats. By placing a significant emphasis on genomics, metabolomics and proteomics-based investigations of plants traits under destructive stresses, the research contributes to the understanding of how plants respond to stresses. The generation of extensive genomics and metabolomics data provides opportunities for the genetics and breeding scientific community to contribute new scientific knowledge to enable plants to cope with climatic changes using the advanced breeding methods. However, the profound challenges posed by climate change demand heightened attention to discover effective strategies for enhancing plant responses to drought, salinity, and heat stresses. The following features are presented as references for advancing plant traits under adverse conditions.

1. Plant diversity coupled with novel plant genomic, transcriptomic, metabolomic and proteomic using data analysis methods that lead to understanding the mechanism of response to destructive stresses.
2. Functional identification and characterization studies on novel stress-responsive genes.
3. Comparative analysis of conventional plant diversity and plant breeding using ‘omics approaches.
4. ‘Omics related traits such as genome-traits, proteome-traits, transcriptome-traits, and metabolome- traits.

## Author contributions

EN: Investigation, Supervision, Writing – original draft. YC: Conceptualization, Formal Analysis, Validation, Writing – review & editing. ME: Data curation, Formal Analysis, Visualization, Writing – review & editing. MA: Methodology, Resources, Software, Writing – review & editing. IE: Data curation, Investigation, Visualization, Writing – review & editing.

